# Challenges in Managing Synchronous Advanced Prostate and Pancreatic Cancers: A Case Report

**DOI:** 10.1159/cro/abzag052

**Published:** 2026-09-08

**Authors:** Fauna Herawati, Kevin Kantono, Taufik Indrawan

**Affiliations:** a Department of Clinical Pharmacy, Faculty of Pharmacy, University of Surabaya, Jalan Raya Kalirungkut, Surabaya, Indonesia; b Department of Food Science, Auckland University of Technology, Auckland, New Zealand; c Urology Clinic, Dr Soewandhie Regional Hospital, Surabaya, Indonesia

**Keywords:** Neoplasms, Multiple primary malignancies, Prostatic neoplasms, Antineoplastic agents, Immunological pancreatic neoplasms, Pancreatic neoplasms, Positron emission tomography-computed tomography

## Abstract

**Introduction:**

Prostate cancer (PCa) and pancreatic cancer (PC) are distinct malignancies with diverging epidemiological and prognostic profiles. PCa often mimics benign prostatic hyperplasia, while PC remains asymptomatic until advanced stages, complicating early detection. As cancer survivorship and screening improve, the incidence of synchronous multiple primary malignancies (MPMs) is rising, with emerging data suggesting a potential biological link between PCa and PC. Managing such cases requires a delicate balance of treatment sequencing and toxicity management. Reporting these rare encounters is vital for refining clinical strategies in complex oncologic presentations.

**Case Presentation:**

This report details the clinical course of a 76-year-old male diagnosed synchronously with high-grade ductal PCa and pancreatic adenocarcinoma. Staging via PSMA PET-CT incidentally identified a pancreatic tail mass with an unusually high SUVmax of 5.2. Despite initiating standard androgen deprivation therapy, the aggressive ductal variant exhibited a “PSA escape” following treatment interruption. As the pancreatic lesion progressed metastatically, the patient’s refusal of conventional chemotherapy and a lack of genomic profiling prompted a salvage, “last-resort” combination of pembrolizumab and olaparib. While this strategy achieved temporary biochemical stabilization and slowed tumor growth, the patient eventually succumbed to metabolic dysregulation and multi-organ failure.

**Conclusions:**

This case highlights the diagnostic challenges of MPM management, specifically rare PSMA-avidity in primary pancreatic tumors which can mimic metastatic spread. While empirical immunotherapy and PARP inhibitor combinations may offer temporary clinical benefit in resource-limited settings, they cannot substitute for biomarker-guided precision medicine. Success depends on strict therapeutic adherence and early multidisciplinary coordination.

## Introduction

Prostate cancer (PCa) and pancreatic cancer (PC) are distinct malignancies with diverging epidemiological and prognostic paths. PCa is the most frequently diagnosed cancer in males globally, whereas PC, though less common, is characterized by its aggressive nature and typically late-stage diagnosis [1–3]. Early detection remains a common hurdle; PCa symptoms often mimic benign conditions, while PC frequently remains asymptomatic until it is metastatic.

Multiple primary malignancies, defined as two or more distinct primary cancers occurring synchronously or metachronously, are increasingly observed due to enhanced screening and prolonged cancer survival [4–6]. Emerging evidence suggests a potential biological link between PCa and PC, possibly mediated by shared genetic susceptibilities such as BRCA2 mutations [7].

The management of synchronous PCa and PC is exceptionally complex due to competing tumor biology and the necessity of balancing treatment sequencing with cumulative toxicity [8–12]. This report describes a unique synchronous presentation where PC was incidentally detected during PCa staging. By highlighting the diagnostic challenges, specifically unusual imaging findings, and the use of an adaptive therapeutic strategy, this case contributes to the limited literature guiding the management of complex oncologic comorbidities.

## Case Presentation

A 76-year-old Asian male presented in 2020 with an enlarged prostate (64.25 mL) and an initial PSA of 19.13 ng/mL. Following a 1-month course of dutasteride and tamsulosin, PSA declined to 8.73 ng/mL (Table 1). Over the subsequent 3 years, PSA levels fluctuated, eventually rising to 16.42 ng/mL by December 2023 despite a lack of urinary symptoms.

**Table 1.** Clinical timeline and biochemical parameters

Timeline	Month 1	Months 3–4	Month 7	Month 8	Month 10	Month 12	Month 15	Month 16	Month 17	Month 17 (late)
Body weight, kg	–	–	75	–	77	75.9	65	62	61	59
Imaging findings	–	FDG-avid lesion at pancreatic tail (SUVmax 3.3; size 1.5 cm)	FDG-avid lesion at pancreatic tail (SUV 7.7; size 1.3 × 1.3 cm)	–	FDG-avid lesion at pancreatic tail (SUV 7.9; size 3.2 × 2.9 cm)	–	–	The current mass size is 8.7 × 4.03 × 3.8 cm	–	Overall dimensions approx. 11.1 × 4.2 × 5.8 cm
Biochemical parameters	Ca 19-9: 26.9 U/mL	Ca 19-9: 39.4 U/mL (month 3)	–	Ca 19-9: 24.3 U/mL	–	Ca 19-9: 42.7 U/mL	Ca 19-9: 799 U/mL	–	Ca 19-9: 3,474.0 U/mL	Ca 19-9: –
						Lipase: 201 U/L	Lipase: 113 U/L		Lipase: 223 U/L	Lipase: 21 U/L
		Ca 19-9: 23.4 U/mL (month 4)		CEA: 1.4 ng/mL		Amylase: 70 U/L	Amylase: 62 U/L		Amylase: 76 U/L	
						CEA: 2.6 ng/mL	CEA: 10.5 ng/mL		CEA: 37.0 ng/mL	Amylase: 34 U/L
HbA1c (%) and metabolic markers	7	–	7.9	Insulin: 1.4 uIU/L	–	8.7	11.8	–	8.6	Patient admitted due to hypoglycemia
						Insulin: 2.6 uIU/L			Insulin: 2.7 uIU/L	
Other laboratory findings	Albumin: 3.9 g/dL	–	Creatinine: 1.03 mg/dL	–	–	Conjugated bilirubin: 0.20 mg/dL	Conjugated bilirubin: 0.38 mg/dL	–	Conjugated bilirubin: 10.20 mg/dL	Conjugated bilirubin: 13.57 mg/dL
						Albumin: 3.9 g/dL	Albumin: 3.8 g/dL		Albumin: 2.9 g/dL	Albumin: 2.2 g/dL
	Creatinine: 1.06 mg/dL									
						Creatinine: –	Creatinine: 0.92 mg/dL		Creatinine: 0.74 mg/dL	Creatinine: 0.68 mg/dL
Treatment	No chemotherapy	No chemotherapy	No chemotherapy	No chemotherapy	No chemotherapy	No chemotherapy	No chemotherapy	Pembrolizumab: 200 mg IV (dose 1)	Pembrolizumab: 200 mg IV (dose 2)	Pembrolizumab: 200 mg IV (dose 3)
									Olaparib: 150 mg bid	Olaparib: 150 mg bid

In early 2024, a suspicious digital rectal examination prompted a 1.5-Tesla multiparametric MRI, which revealed PI-RADS 5 tumor foci in the transitional and peripheral zones. Fusion-guided biopsy (18 cores) confirmed high-grade prostatic adenocarcinoma (Gleason 4 + 5, grade group 5) with a prominent ductal variant characterized by papillary architecture and stratified columnar cells (Fig. 1). The patient was diagnosed with stage III disease and initiated bicalutamide and leuprorelin acetate. Although PSA initially dropped to 0.31 ng/mL, a sharp rebound followed a brief treatment interruption.

**Fig. 1.**
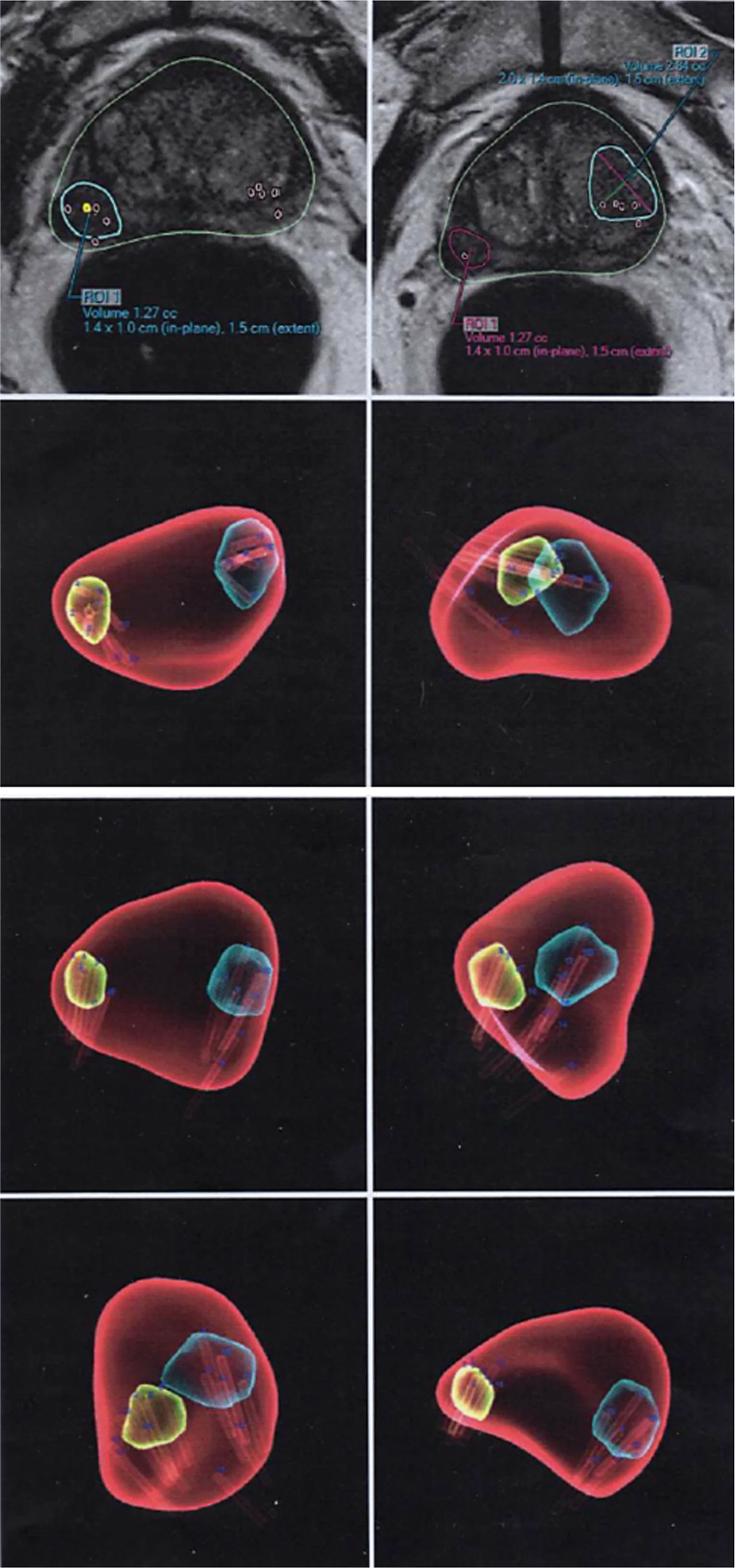
Pre-biopsy multiparametric MRI of the prostate. The top row displays axial T2-weighted sequences with regions of interest (ROIs) identified for targeted sampling (teal and yellow circles). Middle and bottom rows provide three-dimensional volume-rendered reformatting views showing the spatial orientation of the two segmented targeted lesions (yellow and teal) relative to the prostate boundary (semitransparent red). This multi-planar visualization guided the MR/US fusion-guided targeted biopsy to obtain representative tissue from ROI 1 to ROI 2.

Staging with gallium-68 PSMA PET-CT demonstrated intense radiotracer avidity within the primary prostate tumor bed (SUVmax 14.8), with physiological distribution observed in the lacrimal/salivary glands, liver, and kidneys, without evidence of osteoblastic or classic distant prostate metastases. However, it incidentally revealed abnormal uptake in the pancreatic tail (SUVmax 5.2). At this initial detection, the PCa was stage III (Gleason 4 + 5 ductal variant), while the pancreatic mass appeared localized. A curative-intent pathway combining distal pancreatectomy for the pancreatic tail mass and radical prostate radiotherapy, along with endoscopic ultrasound-guided biopsy, was evaluated. However, the patient explicitly declined all surgical resections, invasive diagnostic procedures (including biopsy), and conventional chemotherapy. Consequently, immediate surgical resection could not be pursued; the subsequent interval reflected the patient’s insistence on noninvasive conservative care rather than a planned observation strategy. Serial 18F-FDG PET/CT tracked progressive hypermetabolism in the pancreatic mass: SUVmax 3.3 at months 3–4 (1.5 cm), SUVmax 7.7 at month 7 (1.3 × 1.3 cm), and SUVmax 7.9 at month 10 (3.2 × 2.9 cm). Serial imaging tracked the lesion’s progression from a localized mass to a locally advanced and metastatic 11.1 × 4.2 × 5.8 cm tumor with splenic vessel encasement and liver metastases by April 2025.

The patient declined surgical intervention and conventional chemotherapy. Based on the suspected molecular cross talk between the malignancies, salvage therapy was initiated with pembrolizumab and olaparib without prior biomarker (PD-L1, MSI, or BRCA1/2) confirmation. Following three doses of pembrolizumab and the addition of olaparib, at month 16, treatment response was evaluated over a 4-week window (months 16–17). During this period, serum PSA remained continuously suppressed (<0.5 ng/mL) under androgen deprivation. Concurrently, serum CA 19-9 demonstrated a transient velocity plateau (stabilizing around 3,474.0 U/mL) alongside improvements in clinical performance and glycemic parameters (HbA1c decreasing from 11.8% to 8.6%). Follow-up MRI demonstrated deceleration in volumetric expansion (8.7 × 4.03 × 3.8 cm at month 16 to 11.1 × 4.2 × 5.8 cm at month 17) (Fig. 2), though formal RECIST 1.1 stable disease criteria were not met due to hepatic progression. This temporary clinical and biochemical stabilization lasted approximately 28 days before fulminant disease progression ensued.

**Fig. 2.**
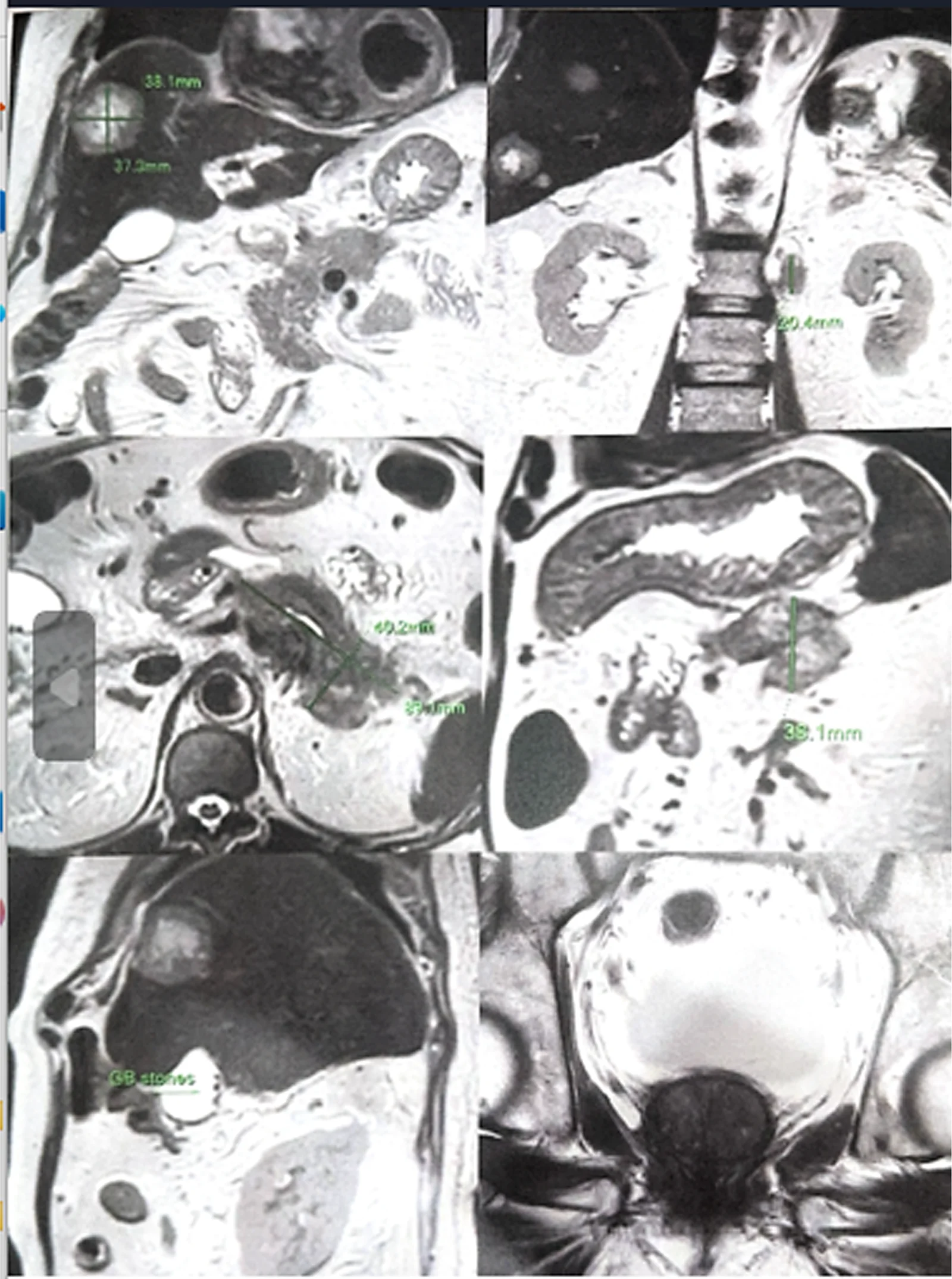
Axial and coronal T2-weighted and post-contrast T1-weighted MRI sequences of the abdomen. The images reveal a large (8.7 × 4.03 × 3.8 cm) heterogeneous mass in the distal pancreatic corpus and tail. The lesion is characterized by spiculated borders, central necrosis, and encasement of the splenic vessels. Additionally, multiple hepatic metastatic nodules are visible in the right lobe (largest measuring 38 mm), accompanied by significant regional and para-aortic lymphadenopathy (largest 2.0 cm).

Five days after the third dose of pembrolizumab, the patient was hospitalized with profound hypoglycemia, hypotension, acute kidney injury, and metabolic acidosis. Despite Intensive Care Unit admission and aggressive vasopressor support, the patient’s condition deteriorated rapidly, leading to a fatal cardiac arrest. The clinical course consisted of multi-organ failure secondary to advanced PC.

## Discussion

The management of synchronous advanced PCa and PC is a formidable clinical challenge, particularly in elderly patients where aggressive ductal histology is present. In this case, the pharmacological synergy of bicalutamide, dutasteride, and leuprorelin provided systemic hormonal suppression [13–16]. While second primary malignancies in PCa survivors are increasingly recognized [4, 6], synchronous presentations of PCa and pancreatic ductal adenocarcinoma (PDAC) remain exceptionally rare in clinical literature [7–9]. Compared to previously reported cases, which predominantly describe standard acinar prostatic adenocarcinomas presenting with metachronous pancreatic lesions [7, 9], our case exhibits three distinct characteristics: (1) aggressive histological presentation featuring a high-grade ductal variant of PCa prone to rapid PSA escape [15, 17]; (2) unique molecular radiotracer overlap, where the pancreatic lesion demonstrated atypical PSMA hypermetabolism (SUVmax 5.2) [10, 18]; and (3) the application of an empirical, off-label salvage combination of pembrolizumab [19] and olaparib [20–22] in an unselected patient, mirroring early evidence from the 2026 POLAR trial [23, 24]. Comparative evaluation of these features against published cohorts underscores the complex diagnostic interplay and aggressive biological behavior inherent to synchronous ductal PCa and PDAC phenotypes [7–10].

A primary diagnostic hurdle in this presentation lies in establishing the precise nature of the dual tumor burden, specifically, determining whether the pancreatic lesion represents a synchronous primary pancreatic adenocarcinoma or an atypical metastatic deposit from the primary ductal PCa (or vice versa) [7, 10]. In clinical practice, distinguishing true multiple primary malignancies from metastatic disease is paramount for appropriate staging and treatment selection. While PSMA PET/CT is standard for PCa staging, its incidental detection of the pancreatic tail mass with an unusually high SUVmax of 5.2 presented a diagnostic dilemma. PCa metastases to the pancreas are exceptionally rare but can demonstrate high PSMA uptake. Conversely, primary PDAC can also exhibit PSMA avidity due to intense expression within the tumor’s neovascular endothelial cells rather than epithelial tumor cells [18, 25]. In this patient, histological confirmation of the pancreatic mass via biopsy was precluded by patient refusal and the clinical imperative to avoid unnecessary procedural risk (non-maleficence) in a fragile, elderly individual. Nevertheless, a synchronous secondary primary PDAC was strongly favored over metastatic PCa based on integrated clinical evidence: (1) distinct biomarker discordance, marked by an exponential rise in CA 19-9 (reaching 3,474 U/mL) during systemic androgen suppression while PSA remained suppressed; (2) rapid, aggressive local invasion with encasement of splenic vessels and progressive liver metastases characteristic of primary PDAC; and (3) clinical manifestation of obstructive jaundice and pancreatic exocrine/endocrine dysfunction [3, 9].

The therapeutic approach used here addresses a common dilemma in resource-limited settings: the “last-resort” use of precision agents without biomarker validation. Although PD-1 inhibition (pembrolizumab) and PARP inhibition (olaparib) usually require MSI-H/dMMR or BRCA1/2 confirmation [23, 26], the observed 4-week period of deceleration in tumor growth velocity and transient CA 19-9 stabilization mirrors emerging results from the 2026 POLAR trial [23]. This suggests a “molecular cross talk” where DNA damage response vulnerabilities can be exploited even in unselected patients when traditional options are refused. However, the patient’s terminal decline, marked by metabolic acidosis and multi-organ failure, underscores the aggressive nature of end-stage PC and the limitations of salvage therapy in the absence of early intervention [27, 28]. This fulminant presentation likely represented a multifactorial confluence of disease-related metabolic failure, underlying comorbidities, and potential treatment-related adverse events. First, from a metabolic and oncologic perspective, end-stage pancreatic adenocarcinoma induces extreme cancer cachexia, extensive hepatic parenchymal replacement by metastases (as evidenced by marked hyperbilirubinemia reaching 13.57 mg/dL), and severe disruption of hepatic gluconeogenesis. Massive pancreatic tissue destruction can also severely disrupt endocrine glucagon/insulin homeostasis. Second, drug-induced toxicities must be critically evaluated. Immune checkpoint inhibitors such as pembrolizumab are well documented to cause rare but life-threatening immune-related adverse events, including acute primary or secondary adrenal insufficiency (hypophysitis) and fulminant autoimmune type 1 diabetes, both of which can precipitate sudden severe hypoglycemia, vascular collapse, and metabolic acidosis. Furthermore, olaparib administration in the context of declining renal function (acute kidney injury) may have led to altered drug clearance, compounding systemic toxicity. Third, the patient’s underlying comorbidities, specifically poorly controlled type 2 diabetes (HbA1c peaking at 11.8%), advanced age (76 years), and progressive sarcopenia (weight loss from 77 kg to 59 kg), marked a precarious physiological baseline highly susceptible to rapid decompensation.

From a therapeutic standpoint, early radical surgical management of the localized pancreatic tail mass combined with prostate radiotherapy represents the optimal curative-intent pathway for fit patients presenting with synchronous localized tumors. In this case, the decision to forgo early surgery and diagnostic biopsy highlights a delicate ethical boundary. Under the principles of beneficence and non-maleficence, clinicians must weigh the curative intent of major surgery against the risk of high perioperative morbidity in an elderly patient. Crucially, ethical practice dictates that patient autonomy remains paramount; the patient’s informed refusal of invasive biopsy, pancreatectomy, and systemic chemotherapy precluded aggressive local management and necessitated noninvasive palliative sequencing. A significant limitation of this report is the lack of molecular profiling and endocrine immune-related adverse event bioassays (such as serum cortisol, ACTH, or C-peptide levels during the hyperacute collapse). While the “treatment attempt out of desperation” yielded a brief clinical reprieve, the authors emphasize that such empirical strategies should not replace standard-of-care diagnostics and require vigilant monitoring for hyperacute metabolic toxicities. Furthermore, the limited involvement of a structured multidisciplinary team likely impacted on the coordination of supportive care during the terminal phase.

## Conclusion

This case demonstrates that while PSMA PET/CT can facilitate the identification of rare synchronous malignancies, differentiating true synchronous primary tumors from metastatic disease remains a critical diagnostic challenge when tissue acquisition is unfeasible. The management of such complex comorbidities in the elderly requires careful integration of imaging and biomarker dynamics to guide care safely and ethically. The temporary response to pembrolizumab and olaparib validates the potential synergy of immunotherapy and PARP inhibition, yet ultimately reinforces that empirical salvage therapies cannot supplant precision medicine-based strategies. Future management of complex synchronous malignancies must prioritize early comprehensive genomic profiling (including BRCA1/2, HRR, and MSI/dMMR status) to guide biomarker-directed targeted therapies, alongside structured multidisciplinary team coordination to optimize clinical durability and patient safety.

## Acknowledgments

The authors would like to thank the patient’s son, for his invaluable technical assistance in meticulously recording all laboratory results, which greatly aided in the data management for this study.

## Statement of Ethics

This case report was conducted in accordance with international ethical guidelines, including the Declaration of Helsinki. This study protocol was reviewed and approved by the Ethics Committee of the University of Surabaya, No. 628/KE/VII/2025 dated July 8, 2025. Informed consent for participation in this study was provided by the patient’s legal guardian. Written informed consent for the publication of relevant clinical details and any accompanying images was obtained from the patient’s legal guardian. The patient’s right to anonymity and privacy has been respected, and no identifying information is included unless deemed essential for scientific purposes. The guardian was informed that personally identifiable material may be available online and in print upon publication. Written informed consent was obtained from the patient’s legal guardian for the publication of this case report and any accompanying images. A copy of the written consent is available for review with the Editor-in-Chief of this journal. This manuscript was prepared following the CARE guidelines (online suppl. Material 1; for all online suppl. material, see https://doi.org/10.1159/cro/abzag052).

## Conflict of Interest Statement

The authors have no conflicts of interest to declare.

## Funding Sources

This study was not supported by any sponsor or funder.

## Author Contributions

F.H.: drafting and finalizing the manuscript, and collecting and describing the patient’s diagnosis and therapeutic course, detailing the clinical presentation, radiologic diagnostics, treatment strategies, and end-of-life management in this case report. K.K. and T.I.: reviewing and editing the manuscript and confirming the authenticity of all the raw data. All authors read and approved the final manuscript.

## Data Availability

The data generated in the present study are not publicly available due to ethical reasons but may be requested from the corresponding author upon reasonable request due to privacy reasons.
